# Analysis of Optical Coherence Tomography (OCT) and Optical Coherence Tomography Angiography (OCTA) Parameters in Young Adults after SARS-CoV-2 Infection (COVID-19) Compared with Healthy Young Controls

**DOI:** 10.3390/diagnostics13071283

**Published:** 2023-03-28

**Authors:** Anahita Bajka, Daniel Rudolf Muth, Maximilian Robert Justus Wiest, Sadiq Said, Magdalena Rejdak, Sophia Sidhu, Nastasia Foa, Frank Blaser, Daniel Barthelmes, Mario Damiano Toro, Eric H. Souied, Jeremy Werner Deuel, Patricia Schlagenhauf, Sandrine Anne Zweifel

**Affiliations:** 1 Department of Ophthalmology, University Hospital Zurich, University of Zurich, 8091 Zurich, Switzerland; 2 Faculty of Medicine, University of California, San Diego, CA 92093, USA; 3 Department of General and Pediatric Ophthalmology, Medical University of Lublin, 20-079 Lublin, Poland; 4 Eye Clinic, Public Health Department, University of Naples Federico II, 80138 Naples, Italy; 5 Department of Ophthalmology, Centre Hospitalier Intercommunal de Creteil, University Paris Est Creteil, 94000 Creteil, France; 6 Department of Global and Public Health, Epidemiology, Biostatistics and Prevention Institute, University of Zurich, 8001 Zurich, Switzerland; 7 Division of Medical Oncology and Haematology, University Hospital of Zurich, 8006 Zurich, Switzerland; 8 MilMedBiol—Centre of Competence for Military Medicine Biology, University of Zurich, 8006 Zurich, Switzerland

**Keywords:** COVID-19, SARS-CoV-2, optical coherence tomography, OCT, optical coherence tomography angiography, OCTA, Long-COVID in military organizations, *LoCoMo*

## Abstract

Purpose: To compare retinal changes in young adults with previous SARS-CoV-2 infection with healthy young controls using optical coherence tomography (OCT) and optical coherence tomography angiography (OCTA). Methods: This prospective single-center study was conducted at the University Hospital of Zurich, Zurich, Switzerland. Participants were imaged from May to November 2021 using the *SOLIX* device (Visionix International SAS, Pont-de-l’Arche, France). We performed 12 mm × 12 mm, 6.4 mm × 6.4 mm, 6 mm × 6 mm and 3 mm × 3 mm OCT and OCTA scans, as well as fundus photography of each participant’s eyes. Results: In total, 466 participants were imaged. Of these, 233 were healthy controls with negative RT-PCR tests for SARS-CoV-2, 168 were young adults who had a SARS-CoV-2 infection at least 180 days previously, 19 were participants who had a SARS-CoV-2 infection < 180 days previously, and 46 were participants with asymptomatic SARS-CoV-2 infection (i.e., serologically positive but with no symptoms). Compared with healthy controls, statistically significant differences were found for OCTA recordings of the optic disc for the whole image (WI) and WI capillary vessel density, with both being higher in the SARS-CoV-2 group. Conclusion: Statistically significant results were only observed for selected variables, and in parts, only unilaterally, with relatively large *p* values (*p* = 0.02–0.03). Thus, we did not interpret these as clinically significant, leading to the conclusion that young and otherwise healthy individuals (mainly men) seem to recover from mild COVID-19 infections with no ophthalmological residues.

## 1. Introduction

Since the outbreak of the COVID-19 pandemic in 2019, substantial research has been performed in different medical fields, including ophthalmology, to analyze the pathophysiological aspects of SARS-CoV-2 and its clinical impact worldwide [1,2,3,4,5,6,7,8,9,10]. From a pathophysiological standpoint, SARS-CoV-2 causes inflammation of the vessels, including vascular leakage and exudation, while thickening the interstitial layer of vessel walls. This leads to a higher risk of thromboembolic events [11,12,13,14,15]. Retinal perfusion occurs in small vessels that are vulnerable to hypercoagulation. Thus, SARS-CoV-2 increases the risk of transient or permanent retinal vessel occlusion, potentially causing long-term changes in the chorioretinal vasculature and its architecture [16,17,18]. The number of confirmed COVID-19 cases is still rising as new variants emerge. Yet, there is a paucity of studies providing information on retinal perfusion and structure in a large cohort of confirmed COVID-19 cases, especially in a young, healthy population [19,20,21,22]. Spectral-Domain Optical Coherence Tomography (SD-OCT) and Optical Coherence Tomography Angiography (OCTA) provide fast and non-invasive methods for analyzing retinal perfusion and are used in clinical settings for routine and follow-up imaging of patients with retinal diseases [23,24,25,26,27]. In this study, we used OCT and OCTA imaging to investigate changes in the structure and perfusion parameters of the retina in a large population of young patients following SARS-CoV-2 infection. These findings were compared with those observed in a group of young healthy controls.

## 2. Materials and Methods

### 2.1. Ethics

Approval for the study was obtained from the local Ethics Committee of the Canton of Zurich (BASEC number 2021-00256).

### 2.2. Study Design

Data presented in this study were obtained as part of the *Long-COVID in Military Organizations* (*LoCoMo*) study. The *LoCoMo* study is a prospective longitudinal cohort study registered with ClinicalTrials.gov (trial number NCT04942249). The participants took part in minimally invasive but intensive battery of tests conducted over a whole day at the University of Zurich and its associated clinics at the University Hospital of Zurich (USZ). All procedures were performed by specially trained qualified physicians, scientists, and study nurses [1].

### 2.3. Participants

Recruits of the Swiss Army serving during the period 2020–2021 were screened. Participants included in the study were army recruits who tested positive or negative for SARS-CoV-2, based on real-time PCR tests, during their army service between 1 March 2020 and 31 December 2020.

Four participant groups were set up: (1) a long post-COVID-19 group with participants who had been diagnosed with SARS-CoV-2 > 180 days prior to study screening; (2) a recent post-COVID-19 group made up of participants who had tested positive for COVID-19 ≤ 180 days; (3) an asymptomatic post-COVID-19 group; (4) a control group with participants who had never been infected with SARS-CoV-2 at the time of study screening.

The status post-COVID-19 was defined as a positive diagnostic RT-PCR test result for SARS-CoV-2 from a commercial provider and concurrent compatible symptoms defined by the Federal Office of Public Health in Switzerland, such as fever, cough, sore throat, malaise, headache, muscle pain, nausea, vomiting, diarrhea, and loss of taste or smell. The day the PCR test was administered was considered the first day of COVID-19 infection. A positive antigen test alone or symptoms alone did not qualify for inclusion in the post-COVID-19 subgroups. Participants not meeting the post-COVID-19 definition but showing a positive serological test for anti-nucleocapsid (Roche Elecsys Anti-SARS-CoV-2; Roche Diagnostics AG, Rotkreuz, Switzerland) were defined as asymptomatic. These participants were assigned to the post-COVID-19 group. Only participants with negative anti-nucleocapsid serology and who did not match the COVID-19 definition were assigned to the control group.

In addition, on the day of the study, a saliva SARS-CoV-2 rapid antigen test was performed to exclude concurrent SARS-CoV-2 infections.

Exclusion criteria were unwillingness to participate or inability to participate on the designated testing day at the testing facility in Zurich, Switzerland.

### 2.4. Data Acquisition

**Figure 1:**
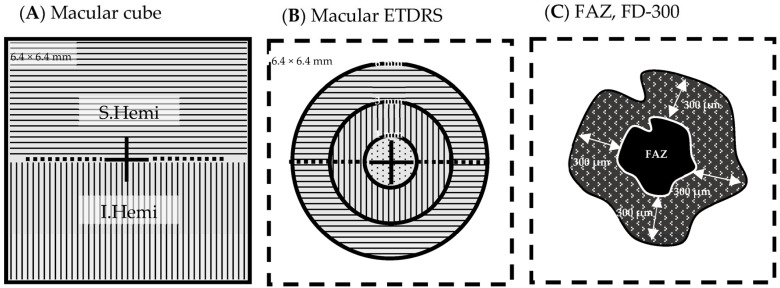
Macular OCT/OCTA cube 6.4 mm × 6.4 mm scan field (**A**) Cube scan analysis parameters: whole image (WI), 6.4 mm × 6.4 mm; superior hemifield (S.Hemi), 3.0 mm × 6.4 mm; and inferior hemifield (I.Hemi), 3.0 mm × 6.4 mm (**B**) ETDRS grid analysis parameters: fovea (ø 0–1 mm), parafovea (ø 1–3 mm), perifovea (ø 3–6 mm), and whole image (6.4 mm × 6.4 mm) (**C**) FAZ area (mm^2^) (homogenous black area), FAZ perimeter (mm) around FAZ (white line), FD-300 vessel density (%) (patterned ring). Legend: ø, diameter; ETDRS, Early Treatment Diabetic Retinopathy Study; FAZ, foveal avascular zone; FD-300, vessel density in a 300 µm wide ring around the FAZ perimeter.

**Figure 2:**
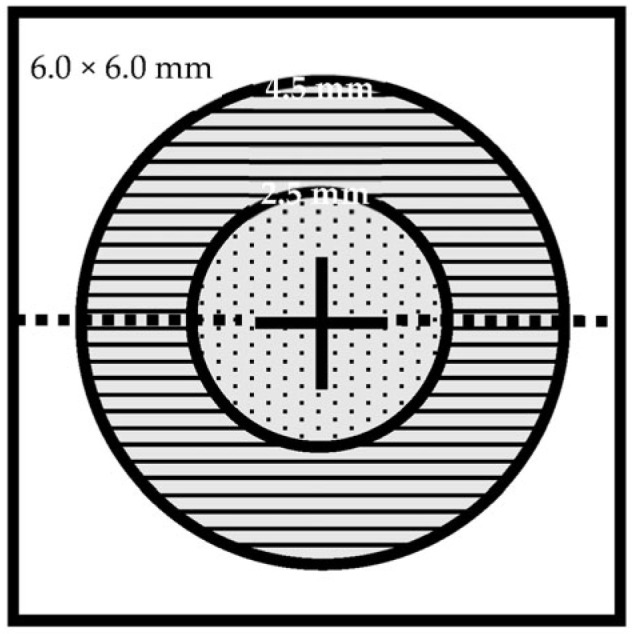
Disc OCT/OCTA cube 6.0 mm × 6.0 mm scan field. Analysis parameters: whole image (WI) (6.0 mm × 6.0 mm), inside disc (ø 2.5 mm), peripapillary (ø 2.5–4.5 mm). Legend: ø, diameter.

**Figure 3:**
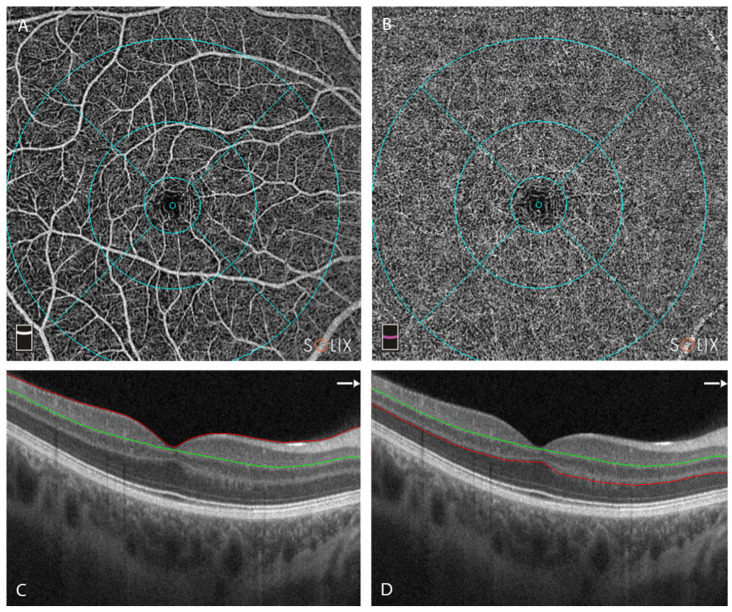
Overview of the optical coherence tomography angiography (OCTA) centered on the fovea. Retinal layer segmentation was automatically performed using the OCTA device and manually reviewed and corrected where necessary. (**A**) En-face image of superficial capillary plexus (SCP) (**B**) En-face image of deep capillary plexus (DCP) (**C**) Cross-sectional OCT B scan centered on the fovea, with segmentation of the SCP. Red line represents upper limit of slab = ILM; green line represents lower limit = IPL − 10 μm [29]. (**D**) Cross-sectional OCT B scan centered on the fovea, with segmentation of the DCP. Green line represents upper limit of slab = IPL − 10 μm; red line represents lower limit = OPL + 10 µm; Legend: ILM, internal limiting membrane; IPL, inner plexiform layer; OPL, outer plexiform layer.

A detailed ophthalmological examination was conducted, including auto-refraction-corrected visual acuity (CVA), intraocular pressure (IOP) measurement, slit lamp examination, biomicroscopy of the anterior segment and the fundus, SD-OCT using the *Solix* device (Visionix International SAS (formerly Optovue), Pont-de-l’Arche, France), and OCTA and fundus photography using the *Solix* device [28]. In addition to the true-color fundus photographs taken using the *Solix* device, fundus imaging using ultra-widefield scanning laser ophthalmoscopy (UWF-SLO) was also conducted using the *California* device (Optos, Marlborough, MA, USA). Qualitative analysis of the UWF-SLO images was conducted by two independent graders (MRJW and TH). CVA was determined from automatic refractory data acquired using an autorefractometer (*NT-530/510*, Nidek Company, Ltd., Gamagori, Japan). The values were then converted to the base 10 logarithm of minimal angle of resolution (logMAR) before further analysis. The same autorefractometer was also used to take non-invasive intraocular pressure (IOP) measurements using air-puff tonometry. Only OCT and OCTA data with quality indexes (QI) ≥ 6/10 were included in the analysis. Scans were obtained using the scan pattern presets in the *Solix* device (Figure 1, Figure 2 and Figure 3). To ensure that comparable anatomic locations were recorded for each participant, each participant was asked to fixate on a built-in target. The correct point of fixation was checked by the person acquiring the images. Retinal layer segmentation was performed automatically using the algorithm built into the OCT device. The accuracy of the segmentation was reviewed and manually corrected where necessary.

**Figure 4:**
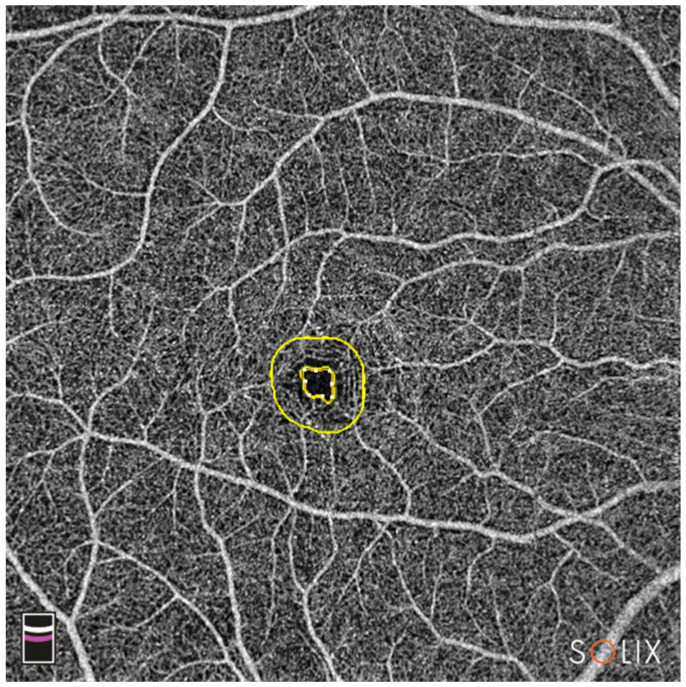
En-face OCTA image of the combined SCP and DCP. The inner yellow circle marks the outer border of the FAZ; the outer yellow circle represents 300 µm from the FAZ border; FD-300 vessel density between the rings is calculated by the device. Legend: DCP, deep capillary plexus; FAZ, foveal avascular zone; OCTA, optical coherence tomography angiography; SCP, superficial capillary plexus.

- OCT (structural):
  ○ Macula cube scan (6.4 mm × 6.4 mm): Retinal thickness (THK) from internal limiting membrane (ILM) to Bruch’s membrane (BM), retinal volume (VOL) from ILM to BM
  ○ Optic disc cube scan (6 mm × 6 mm): Retinal nerve fiber layer (RNFL) rim THK from ILM to RNFL, rim VOL
- OCTA:
  ○ Macula cube scan (6.4 mm × 6.4 mm) vessel density:
    ⯀ Superficial retinal capillary plexus (SRCP) (Figure 3A,C):
      - whole image (WI) (6.4 mm × 6.4 mm) (%)
      - whole image superior hemifield (S.Hemi) (3.0 mm × 6.4 mm) (%)
      - whole image inferior hemifield (I.Hemi) (3.0 mm × 6.4 mm) (%)
      - ETDRS whole image (0–6 mm diameter) (%)
      - ETDRS fovea (0–1 mm diameter circle) (%)
      - ETDRS parafovea whole (1–3 mm diameter ring) (%)
        ○ parafovea S.Hemi (%)
        ○ parafovea I.Hemi (%)
      - ETDRS perifovea whole (3–6 mm diameter ring) (%)
        ○ parafovea S.Hemi (%)
        ○ parafovea I.Hemi (%)
      - Foveal avascular zone (FAZ) area (mm^2^) calculation
      - FAZ perimeter (mm) calculation
      - FD.300: vessel density of the 300 µm ring surrounding the FAZ (%) (Figure 4)
    ⯀ Deep retinal capillary plexus (DRCP) (Figure 3B,D): same parameters as for SRCP.
  ○ Optic disc cube scan (6 mm × 6 mm): radial peripapillary capillaries (RPC) for the whole image all vessels, whole image capillary, inside disc all vessels, inside disc capillary, peripapillary all vessels, peripapillary capillary.

In line with the current literature, the output value of the vessel density (VD) was used for OCTA analysis [28,30,31,32,33,34]. Additional data, including sex, age on the date of examination, and COVID-19 exposure, were recorded.

### 2.5. Statistical Analysis

**Figure 5:**
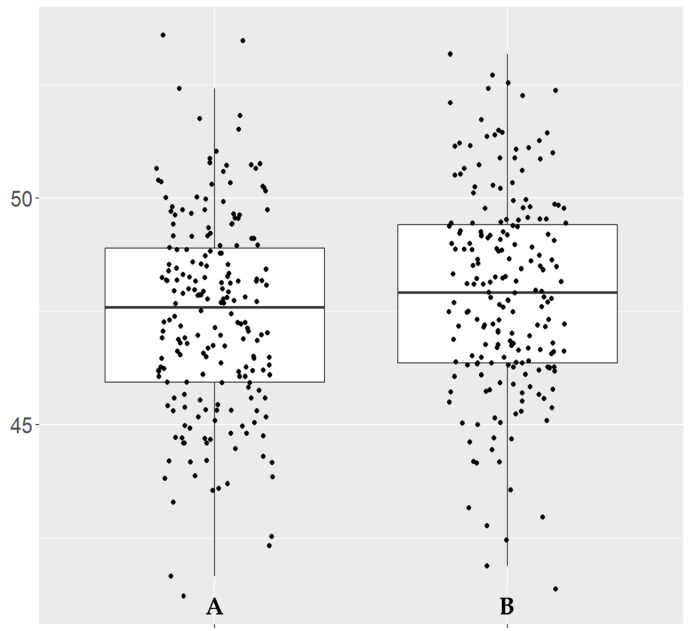
Box plot of OD__OCTA_Disc_6×6_RPC__Whole.Image.Capillary vessel density (%). (**A**) Control group. (**B**) COVID-19 group.

Data were organized in Excel (Microsoft Corp., Redmond, WA, USA) and analyzed using R.app (v4.1.0 GUI 1.76 for MacOS, The R Foundation for Statistical Computing c/o Institute for Statistics and Mathematics, Vienna, Austria), RStudio (2022.02.3 Build 492 for MacOS, Rstudio PBC, Boston, MA, USA) and StatPlus:mac (v8.0.1.0 for MacOS, AnalystSoft, Walnut, CA, USA). Descriptive statistics, including arithmetic mean and standard deviation (SD) and median (min, max, range) were computed (Table 1). Two subgroups were defined for further statistical analysis: (1) an all-COVID-19 group that included participants from the long post-COVID-19 and the recent post-COVID-19 subgroups; (2) a control group defined as above (see the section on participants). The Wilcoxon rank sum test was calculated to evaluate differences between the all-COVID-19 and control groups. Odds ratios (ORs) were computed as predictive values [35]. In our case, an OR of 1 meant that the respective variable could statistically not be used to discriminate between the healthy control group and the post-COVID-19 group. This was also the case if the 95% confidence interval (95% CI) encompassed the value 1 (see Figure 5). An OR > 1 with a 95% CI not including 1 meant that the corresponding parameter favored the post-COVID-19 group, while an OR < 1 with an associated 95% CI favored the control group. Only OCT and OCTA data with quality indexes ≥ 6/10 were analyzed. Image-quality grading was performed automatically using the OCT/OCTA device (Solix). The statistical significance level (α) for all tests was set at 0.05. Results with a *p* value less than 0.05 (*p* < 0.05) were interpreted as statistically significant.

## 3. Results

Detailed demographic data are provided in Table 1.

Green and red laser UWF ophthalmoscopy (SLO) and autofluorescence (AF) images from both eyes of 466 study participants were available. Of the 932 eyes, 616 (66.09%) were classified as “unremarkable” based on available imaging data. A total of 26 eyes (5.58%) showed peripheral degenerative alterations, 34 (7.3%) showed “other retinal findings”, 85 (18.24%) showed retinal vessel tortuosity, 18 (3.86%) showed optic nerve head anomalies, and 10 (2.15%) showed “other optic nerve head findings”. No cotton wool spots, hemorrhages, or Hollenhorst plaques were observed in any of the 932 eyes.

To evaluate the possible effects of COVID-19 infection on retinal structure and retinal microvasculature, we computed the Wilcoxon rank sum test for the structural OCT and OCT angiography parameters between healthy controls and all COVID-19-infected (serologically positive) individuals (Table 2).

Compared with healthy controls, statistically significant differences were only observed for OCTA recordings of the disc for the whole image (WI) vessel density and WI capillary vessel density, which were higher in the COVID-19 group (Table 2). However, the significant changes were only observed for the right eye (OD) and for the average of both eyes (OU) (Table 2). To represent the statistically significant values in our study, we generated a box plot for the parameter with the lowest *p*-value (OD__OCTA_Disc_6×6_RPC__Whole.Image.Capillary, *p* = 0.021), showing the distribution pattern for this single parameter (Figure 5).

**Figure 6:**
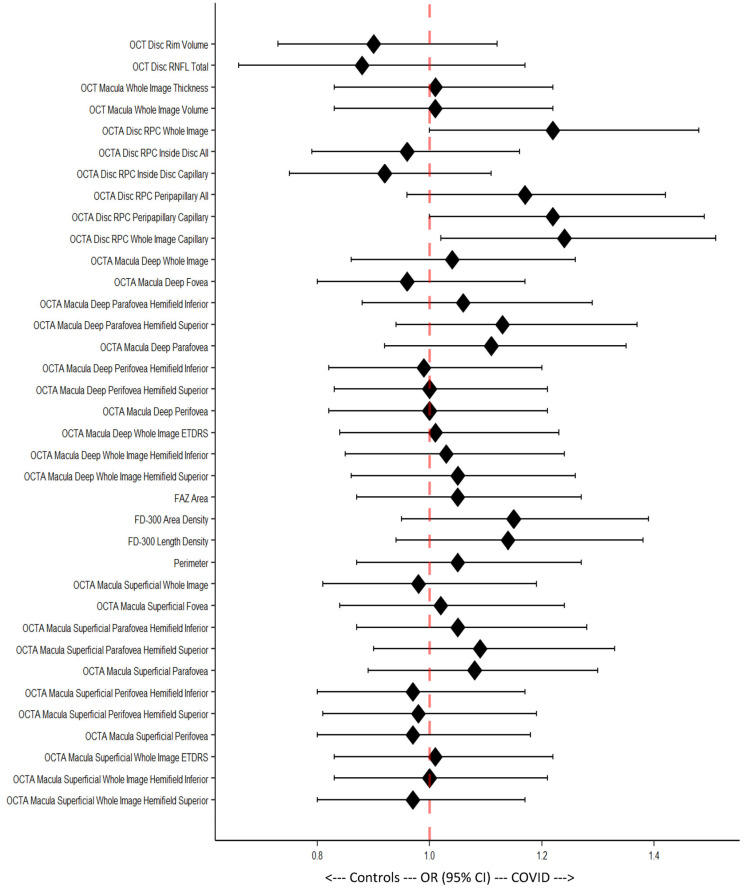
Forest plot of Odds ratios (ORs) with 95% confidence intervals (95% CIs). Each 95% CI that includes the middle line (dotted, red) is considered statistically non-significant.

The OD odds ratios (ORs) were calculated and graphically illustrated using a forest plot (Figure 6). The figure shows results corresponding to the Wilcoxon rank sum test of the OCTA disc WI (*p* = 0.047), WI capillary (*p* = 0.033), and peripapillary capillary (*p* = 0.045), with only vessel density having statistically significant values (Figure 6).

## 4. Discussion

We compared OCTA images of young healthy controls with those of young adults who had previous SARS-CoV-2 infections. Our data showed sporadic, mostly unilateral, weak statistically significant changes only in OCTA optic disc measurements between the groups. We therefore did not consider them clinically significant as majority of the evaluated parameters were statistically non-significant.

There are numerous reports in the literature about structural retinal changes triggered by SARS-CoV-2 (COVID-19), for example paracentral acute middle maculopathy (PAMM) [36,37,38]. Assuming that PAMM and acute macular neuroretinopathy (AMN) are deficits in the retinal capillary plexus, OCTA should provide a clearer picture when compared with uninfected controls. Evaluating post-hospitalized COVID-19 patients who needed partial supportive oxygen and ventilation therapy, Savastano et al. found significantly higher prevalence of systemic disorders such as arterial hypertension and diabetes mellitus [39]. They also identified a negative linear correlation between body mass index (BMI) and OCTA perfusion parameters in the COVID-19 group that was not present in the control group [39]. Abrishami et al. reported significantly lower vessel density (VD) in both the superficial and deep retinal capillary plexus at least two weeks after recovery from systemic COVID-19 symptoms [40]. Furthermore, they stated that the VD seemed to be influenced by disease severity (hospitalized vs. non-hospitalized) [40]. In support of this theory, mean vein diameter (MVD) has been correlated with COVID-19 severity [41]. In contrast to our study population, Savastano et al. and Invernizzi et al. reported retinal cotton wool spots (CWS), retinal hemorrhages, dilated veins, and tortuous vessels in the COVID-19 group. Invernizzi et al. studied participants within 30 days of symptom onset—while Savastano et al. studied participants at least one month after discharge from hospital [39,41]. Very little to none of such findings were found in their respective control groups [39,41]. These findings highlight that patients who were more severely affected by COVID-19 presented visible retinal pathologies after recovering from systemic symptoms. We did not detect similar retinal alterations in our COVID-19 group due to lower severity of the COVID-19 infections, the characteristics of our participants (young, physically trained in the army), and the longer recovery period (>180 days) of the majority of the evaluated post-COVID-19 individuals. This supports the theory that retinal changes occur mainly in the acute phase of COVID-19 infection. Structurally, the retinal findings show a restoration of the systemic rehabilitation phase to a level that no permanent long-term effects are reproducibly detectable using current imaging methods [39]. More severe COVID-19 infection might prolong retinal rehabilitation [34]. Functionally, based on current knowledge, the retina does not seem to be (permanently) affected by the current COVID-19 variants.

Not surprisingly, the number of publications in PubMed (www.pubmed.gov) has grown almost exponentially over the last three years since COVID-19 was first described (Table 3). Looking through the most recent publications shows that there are reports of statistically significant OCTA changes within the macular region and peripapillary of post-COVID-19 patients compared with healthy controls (Table 4). The OCTA parameters that were found to be significant in the majority of the studies are the same as the parameters we evaluated in our study. However, with only a single statistically significant measure and *p*-values ≥0.02, we are uncertain about the clinical significance of the differences observed in our study. Bearing in mind that the COVID-19 group in our study was four to five times larger than previously reported cohorts, and that we had a well-matched serologically negative control group, we expected to see clear and bilateral changes. We have, therefore, interpreted our values as non-significant. 

We acknowledge that our study has some limitations, including the cohorts consisting of only young, predominantly male participants who had suffered mainly from mild SARS-CoV-2 infection. This may have impeded comparability with results from other studies. Therefore, future studies will focus on recruiting an age-matched female cohort to rule out sex-based confounders. Based on findings of sex-based differences, further studies analyzing COVID-19 sequelae may be designed.

## 5. Conclusions

It has been shown that sequelae from mild COVID-19 infections, including chronic fatigue syndrome (CFS), hyposmia, psychological deficits, and reduced cardiovascular status, can persist for months [1]. Although data on ocular COVID sequelae is still lacking in homogeneity, it seems that young and otherwise healthy individuals recover from mild COVID-19 infections with no detectable ophthalmological residues.

## Figures and Tables

**Table 1:** Demographic data.

	Long Post-COVID-19 (>180 d)	Recent Post- COVID-19 (≤180 d)	Asymptomatic Post-COVID-19	Control Group (SARS-CoV-2 Negative)	*p*
Group size (*n*)	168	19	46	233	N/A
COVID vaccination status: At least 1 SARS-CoV-2 vaccine prior to study	118 (70%)	10 (53%)	30 (65%)	191 (82%)	N/A
Time (d) from COVID diagnosis to study start (median (25%; 75% quantile)	317 (272; 414)	101 (83; 155)	N/A	N/A	N/A
Age (years) (mean ± SD (95% CI) | median)	22.5 ± 2.5 (22.1; 22.9) | 22	21.3 ± 1.2 (20.8; 21.9) | 21	21.8 ± 2.0 (21.2; 22.4) | 21	21.7 ± 1.6 (21.5; 21.9) | 21	0.07423
Sex ratio female:male	8 (5%):160 (95%)	1 (5%):18 (95%)	3 (7%):43 (93%)	13 (6%):220 (94%)	N/A
Roche Anti-SARS-CoV-2 test cobas.S (mean ± SD (95% CI))	185.33660 ± 96.30346 (170.66782; 200.00538)	172.30579 ± 94.23907 (126.88401; 217.72757)	207.35870 ± 78.80898 (183.95532; 230.76207)	178.79528 ± 108.96684 (164.73040; 192.86016)	0.60617
Roche Anti-SARS-CoV-2 test cobas.N (mean ± SD (95% CI))	8.58670 ± 15.09939 (6.28678; 10.88661)	28.70184 ± 43.61160 (7.68173; 49.72196)	8.88696 ± 16.08435 (4.11049; 13.66342)	0.30860 ± 2.02638 (0.04704; 0.57015)	<0.00001 *
CVA OD (logMAR) (mean ± SD (95% CI))	−0.09 ± 0.08 (−0.10; −0.08)	−0.08 ± 0.08 (−0.08 ± 0.08)	−0.06 ± 0.07 (−0.09; −0.04)	−0.08 ± 0.08 (−0.09; −0.07)	0.18882
CVA OS (logMAR) (mean ± SD (95% CI))	−0.10 ± 0.08 (−0.11; −0.08)	−0.08 ± 0.09 (−0.12; −0.04)	−0.09 ± 0.10 (−0.12; −0.06)	−0.08 ± 0.10 (−0.09; −0.06)	0.53837
IOP OD (mmHg) (mean ± SD (95% CI))	14.7 ± 2.5 (14.3; 15.1)	14.5 ± 2.4 (13.4; 15.7)	14.9 ± 2.9 (14.0; 15.7)	14.8 ± 2.8 (14.4; 15.1)	0.92173
IOP OS (mmHg) (mean ± SD (95% CI))	14.8 ± 2.8 (14.3; 15.2)	15.3 ± 3.1 (13.8; 16.8)	14.8 ± 2.8 (13.9; 15.6)	15.4 ± 8.1 (14.3; 16.4)	0.85845
SE OD (D) (mean ± SD (95% CI))	−0.49 ± 1.34 (−0.70; −0.29)	−0.22 ± 1.37 (−0.88; 0.45)	−0.86 ± 2.05 (−1.47; −0.25)	−0.49 ± 1.34 (−0.67; −0.32)	0.82620
SE OS (D) (mean ± SD (95% CI))	−0.48 ± 1.43 (−0.70; −0.26)	−0.70 ± 1.65 (−1.49; 0.10)	−0.88 ± 1.93 (−1.45; −0.30)	−0.47 ± 1.30 (−0.64; −0.30)	0.76287
Smoking status: Never Ex-smoker Active smoker Unknown	108 (64%) 20 (12%) 33 (20%) 7 (4%)	12 (63%) 0 (0%) 7 (37%) 0 (0%)	30 (65%) 3 (7%) 11 (24%) 2 (4%)	158 (68%) 13 (6%) 51 (22%) 11 (5%)	0.88565
Body height (cm) (mean ± SD (95% CI))	179.16 ± 11.58 (177.09; 181.24)	179.71 ± 9.02 (174.51; 184.92)	178.63 ± 6.79 (176.18; 181.07)	178.77 ± 7.47 (177.73; 179.81)	0.82543
Body weight (kg) (mean ± SD (95% CI))	79.21 ± 15.81 (76.38 82.05)	74.50 ± 11.47 (67.88; 81.12)	79.55 ± 16.85 (73.47; 85.62)	75.63 ± 11.48 (74.04; 77.23)	0.21742

Legend: 95% CI, 95% confidence interval; CVA, corrected visual acuity (auto refractor); D, diopters; IOP, intraocular pressure; logMAR, base 10 logarithm of minimal angle of resolution; N/A, not applicable; min, minimum; max, maximum; OD, oculus dexter, OS, oculus sinister; *p*, statistical *p*-value of Kruskal Wallis test, considered significant when *p* < 0.05 (*); SD, standard deviation of arithmetic mean; SE, spherical equivalent.

**Table 2:** Wilcoxon rank sum test results.

	Control Group (Mean (95% CI))	All-COVID-19 Group (Mean (95% CI))	*p*-Value
**OCTA quality index (QI)**			
OD__OCTA_Macula_6.4×6.4__Quality.Index.QI	9.00 (6.00;10.0)	9.00 (6.00;10.0)	0.845
OS__OCTA_Macula_6.4×6.4__Quality.Index.QI	9.00 (6.00;10.0)	9.00 (6.00;10.0)	0.800
**OCTA MACULA superior retinal capillary plexus (SRCP) vessel density (%)**			
OD__OCTA_Macula_6.4×6.4__SRCP_Whole.Image	49.6 (43.0;51.8)	49.5 (43.7;52.1)	0.495
OD__OCTA_Macula_6.4×6.4__SRCP_Whole.Image.S.Hemi	49.6 (42.4;53.7)	49.6 (43.9;51.9)	0.722
OD__OCTA_Macula_6.4×6.4__SRCP_Whole.Image.I.Hemi	49.7 (43.7;52.3)	49.5 (43.3;52.4)	0.302
OD__OCTA_Macula_6.4×6.4__SRCP_Whole.ETDRS	49.3 (41.9;51.6)	49.2 (42.9;51.9)	0.462
OD__OCTA_Macula_6.4×6.4__SRCP_Fovea	31.2 (15.7;40.2)	31.2 (12.8;42.9)	0.965
OD__OCTA_Macula_6.4×6.4__SRCP_ParaFovea	51.1 (37.7;54.6)	50.9 (42.0;54.0)	0.509
OD__OCTA_Macula_6.4×6.4__SRCP_Para.S.Hemi	51.0 (37.5;54.7)	51.2 (41.6;53.9)	0.952
OD__OCTA_Macula_6.4×6.4__SRCP_Para.I.Hemi	51.1 (37.9;55.2)	50.8 (41.1;54.8)	0.189
OD__OCTA_Macula_6.4×6.4__SRCP_PeriFovea	49.4 (43.9;52.0)	49.3 (43.9;52.0)	0.488
OD__OCTA_Macula_6.4×6.4__SRCP_Peri.S.Hemi	49.4 (42.9;52.6)	49.4 (43.0;51.9)	0.750
OD__OCTA_Macula_6.4×6.4__SRCP_Peri.I.Hemi	49.6 (44.4;52.4)	49.3 (43.6;52.6)	0.314
OS__OCTA_Macula_6.4×6.4__SRCP_Density.Whole.Image	49.6 (43.7;52.0)	49.5 (43.5;52.1)	0.973
OS__OCTA_Macula_6.4×6.4__SRCP_Whole.Image.S.Hemi	49.7 (43.0;52.2)	49.5 (43.8;52.0)	0.775
OS__OCTA_Macula_6.4×6.4__SRCP_Whole.Image.I.Hemi	49.5 (44.5;52.4)	49.6 (43.2;52.5)	0.706
OS__OCTA_Macula_6.4×6.4__SRCP_Whole.ETDRS	49.1 (43.0;51.7)	49.2 (42.8;51.9)	0.767
OS__OCTA_Macula_6.4×6.4__SRCP_Fovea	31.2 (15.8;40.4)	31.2 (17.0;42.0)	0.932
OS__OCTA_Macula_6.4×6.4__SRCP_ParaFovea	50.6 (39.6;54.4)	50.9 (39.5;54.7)	0.259
OS__OCTA_Macula_6.4×6.4__SRCP_Para.S.Hemi	50.7 (40.9;54.8)	50.8 (39.2;55.0)	0.209
OS__OCTA_Macula_6.4×6.4__SRCP_Para.I.Hemi	50.7 (38.3;54.1)	50.7 (39.9;54.8)	0.453
OS__OCTA_Macula_6.4×6.4__SRCP_PeriFovea	49.3 (44.4;51.8)	49.3 (44.1;51.9)	0.937
OS__OCTA_Macula_6.4×6.4__SRCP_Peri.S.Hemi	49.1 (43.1;52.3)	49.3 (43.0;52.2)	0.987
OS__OCTA_Macula_6.4×6.4__SRCP_Peri.I.Hemi	49.3 (44.4;52.5)	49.4 (43.3;52.2)	0.968
OU__OCTA_Macula_6.4×6.4__SRCP_Whole.Image	49.5 (43.0;51.6)	49.5 (45.8;51.6)	0.781
OU__OCTA_Macula_6.4×6.4__SRCP_Whole.Image.S.Hemi	49.4 (42.4;52.1)	49.5 (45.4;51.9)	0.740
OU__OCTA_Macula_6.4×6.4__SRCP_Whole.Image.I.Hemi	49.3 (43.7;51.8)	49.4 (44.8;51.7)	0.901
OU__OCTA_Macula_6.4×6.4__SRCP_Whole.ETDRS	49.1 (41.9;51.6)	49.2 (44.3;51.3)	0.974
OU__OCTA_Macula_6.4×6.4__SRCP_Fovea	31.1 (18.7;40.2)	31.0 (16.9;42.4)	0.955
OU__OCTA_Macula_6.4×6.4__SRCP_ParaFovea	50.7 (37.7;53.9)	50.8 (42.0;53.5)	0.553
OU__OCTA_Macula_6.4×6.4__SRCP_Para.S.Hemi	50.7 (37.5;53.9)	50.8 (42.8;54.2)	0.346
OU__OCTA_Macula_6.4×6.4__SRCP_Para.I.Hemi	50.8 (37.9;54.1)	50.6 (41.2;54.5)	0.935
OU__OCTA_Macula_6.4×6.4__SRCP_PeriFovea	49.3 (43.9;51.7)	49.3 (45.5;51.5)	0.807
OU__OCTA_Macula_6.4×6.4__SRCP_Peri.S.Hemi	49.2 (42.9;52.0)	49.3 (45.0;51.8)	0.967
OU__OCTA_Macula_6.4×6.4__SRCP_Peri.I.Hemi	49.3 (45.0;52.2)	49.2 (44.8;51.6)	0.675
**OCTA MACULA deep retinal capillary plexus (DRCP) vessel density (%)**			
OD__OCTA_Macula_6.4×6.4__DRCP_Density.Whole.Image	54.1 (46.7;55.4)	54.1 (47.1;55.6)	0.814
OD__OCTA_Macula_6.4×6.4__DRCP_Whole.Image.S.Hemi	53.9 (47.3;55.7)	54.0 (45.5;56.2)	0.703
OD__OCTA_Macula_6.4×6.4__DRCP_Whole.Image.I.Hemi	54.2 (45.0;55.8)	54.2 (44.0;55.9)	0.803
OD__OCTA_Macula_6.4×6.4__DRCP_Whole.ETDRS	54.0 (48.9;55.6)	54.0 (48.1;55.8)	0.781
OD__OCTA_Macula_6.4×6.4__DRCP_Fovea	31.1 (14.7;45.3)	31.5 (11.6;46.0)	0.732
OD__OCTA_Macula_6.4×6.4__DRCP_ParaFovea	54.7 (51.3;57.1)	54.8 (50.2;57.3)	0.496
OD__OCTA_Macula_6.4×6.4__DRCP_Para.S.Hemi	54.5 (50.2;57.4)	54.6 (49.8;57.4)	0.683
OD__OCTA_Macula_6.4×6.4__DRCP_Para.I.Hemi	54.8 (50.5;58.0)	55.0 (49.2;57.8)	0.538
OD__OCTA_Macula_6.4×6.4__DRCP_PeriFovea	54.5 (48.7;56.6)	54.5 (47.6;56.8)	0.859
OD__OCTA_Macula_6.4×6.4__DRCP_Peri.S.Hemi	54.4 (49.3;56.9)	54.5 (48.2;57.4)	0.336
OD__OCTA_Macula_6.4×6.4__DRCP_Peri.I.Hemi	54.5 (46.9;56.9)	54.5 (42.9;56.7)	0.652
OD__OCTA_Macula_6.4×6.4__FD.300.Area.Density	51.8 (33.5;59.5)	52.2 (40.6;59.7)	0.957
OD__OCTA_Macula_6.4×6.4__FD.300.Length.Density	17.3 (14.4;19.0)	17.3 (14.7;18.9)	0.625
OS__OCTA_Macula_6.4×6.4__DRCP_Density.Whole.Image	54.0 (42.0;55.9)	53.9 (46.6;55.9)	0.733
OS__OCTA_Macula_6.4×6.4__DRCP_Whole.Image.S.Hemi	53.9 (41.6;55.9)	53.9 (45.7;56.0)	0.489
OS__OCTA_Macula_6.4×6.4__DRCP_Whole.Image.I.Hemi	53.9 (42.5;56.0)	53.9 (46.3;56.0)	0.717
OS__OCTA_Macula_6.4×6.4__DRCP_Whole.ETDRS	53.8 (44.6;56.1)	53.8 (48.9;56.2)	0.535
OS__OCTA_Macula_6.4×6.4__DRCP_Fovea	31.6 (17.4;46.5)	31.7 (14.9;45.0)	0.773
OS__OCTA_Macula_6.4×6.4__DRCP_ParaFovea	54.6 (50.1;57.1)	54.8 (50.5;56.6)	0.331
OS__OCTA_Macula_6.4×6.4__DRCP_Para.S.Hemi	54.5 (49.9;57.3)	54.7 (50.6;57.7)	0.234
OS__OCTA_Macula_6.4×6.4__DRCP_Para.I.Hemi	54.7 (49.9;58.0)	54.7 (49.3;57.7)	0.829
OS__OCTA_Macula_6.4×6.4__DRCP_PeriFovea	54.4 (43.7;56.8)	54.3 (48.4;57.0)	0.336
OS__OCTA_Macula_6.4×6.4__DRCP_Peri.S.Hemi	54.5 (44.6;57.0)	54.2 (47.8;57.3)	0.116
OS__OCTA_Macula_6.4×6.4__DRCP_Peri.I.Hemi	54.4 (42.7;56.8)	54.4 (43.5;56.8)	0.900
OS__OCTA_Macula_6.4×6.4__FD.300.Area.Density	51.6 (35.7;59.4)	52.2 (33.8;59.7)	0.085
OS__OCTA_Macula_6.4×6.4__FD.300.Length.Density	17.1 (14.4;19.0)	17.1 (14.9;18.9)	0.330
OU__OCTA_Macula_6.4×6.4__DRCP_Density.Whole.Image	53.8 (46.7;55.3)	54.0 (48.9;55.5)	0.694
OU__OCTA_Macula_6.4×6.4__DRCP_Whole.Image.S.Hemi	53.7 (46.7;55.5)	53.9 (48.1;55.5)	0.679
OU__OCTA_Macula_6.4×6.4__DRCP_Whole.Image.I.Hemi	53.9 (45.0;55.2)	53.9 (49.0;55.5)	0.752
OU__OCTA_Macula_6.4×6.4__DRCP_Whole.ETDRS	53.8 (49.1;55.2)	53.8 (50.2;55.3)	0.919
OU__OCTA_Macula_6.4×6.4__DRCP_Fovea	31.2 (16.0;46.5)	31.4 (14.0;43.8)	0.834
OU__OCTA_Macula_6.4×6.4__DRCP_ParaFovea	54.6 (51.4;56.5)	54.7 (51.6;56.5)	0.194
OU__OCTA_Macula_6.4×6.4__DRCP_Para.S.Hemi	54.4 (50.9;56.6)	54.5 (51.8;56.5)	0.179
OU__OCTA_Macula_6.4×6.4__DRCP_Para.I.Hemi	54.7 (51.0;57.0)	54.7 (51.1;56.8)	0.467
OU__OCTA_Macula_6.4×6.4__DRCP_PeriFovea	54.3 (48.7;55.9)	54.3 (49.4;56.4)	0.844
OU__OCTA_Macula_6.4×6.4__DRCP_Peri.S.Hemi	54.2 (49.7;56.0)	54.3 (50.8;56.7)	0.970
OU__OCTA_Macula_6.4×6.4__DRCP_Peri.I.Hemi	54.3 (46.9;56.0)	54.3 (47.7;56.0)	0.840
OU__OCTA_Macula_6.4×6.4__FD.300.Area.Density	51.6 (33.5;57.8)	52.0 (43.3;58.5)	0.263
OU__OCTA_Macula_6.4×6.4__FD.300.Length.Density	17.2 (14.7;18.8)	17.2 (15.6;18.7)	0.247
**OCTA DISC radial peripapillary capillary (RPC)**			
OD__OCTA_Disc_6×6_RPC__Density.Whole.Image	54.6 (48.2;59.7)	55.0 (48.4;60.3)	0.034 *
OD__OCTA_Disc_6×6_RPC__Whole.Image.Capillary	47.6 (41.2;53.6)	47.9 (41.4;53.2)	0.021 *
OD__OCTA_Disc_6×6_RPC__Inside.Disc.All	65.6 (47.1;82.2)	65.6 (37.8;79.1)	0.618
OD__OCTA_Disc_6×6_RPC__Inside.Disc.Capillary	55.5 (28.9;75.5)	55.5 (21.5;75.4)	0.235
OD__OCTA_Disc_6×6_RPC__Peripapillary.All	55.9 (47.3;60.7)	56.2 (48.9;61.1)	0.163
OD__OCTA_Disc_6×6_RPC__Peripapillary.Capillary	48.3 (42.0;54.8)	48.6 (43.2;57.2)	0.099
OS__OCTA_Disc_6×6_RPC__Density.Whole.Image	54.5 (45.9;58.6)	54.8 (49.9;61.3)	0.326
OS__OCTA_Disc_6×6_RPC__Whole.Image.Capillary	47.4 (37.1;54.3)	47.8 (42.2;56.4)	0.240
OS__OCTA_Disc_6×6_RPC__Inside.Disc.All	65.4 (41.8;78.3)	65.4 (31.1;84.8)	0.854
OS__OCTA_Disc_6×6_RPC__Inside.Disc.Capillary	54.5 (26.3;71.8)	55.1 (17.9;73.0)	0.953
OS__OCTA_Disc_6×6_RPC__Peripapillary.All	55.6 (47.2;60.6)	56.0 (48.0;61.8)	0.308
OS__OCTA_Disc_6×6_RPC__Peripapillary.Capillary	48.2 (39.1;53.3)	48.6 (43.0;58.1)	0.257
OU__OCTA_Disc_6×6_RPC__Density.Whole.Image	54.6 (47.7;58.9)	55.0 (50.2;58.6)	0.071
OU__OCTA_Disc_6×6_RPC__Whole.Image.Capillary	47.6 (39.2;52.6)	48.1 (42.6;52.3)	0.031 *
OU__OCTA_Disc_6×6_RPC__Inside.Disc.All	65.2 (46.4;74.9)	65.0 (31.1;84.8)	0.738
OU__OCTA_Disc_6×6_RPC__Inside.Disc.Capillary	55.1 (33.5;66.4)	54.5 (17.9;73.0)	0.321
OU__OCTA_Disc_6×6_RPC__Peripapillary.All	55.9 (49.0;60.0)	56.2 (48.5;59.3)	0.134
OU__OCTA_Disc_6×6_RPC__Peripapillary.Capillary	48.4 (40.7;53.4)	48.7 (43.3;53.7)	0.093
**OCTA MACULA foveal avascular zone (FAZ, mm^2^) and perimeter (mm)**			
OD__OCTA_Macula_6.4×6.4__FAZ.Area	0.21 (0.04;0.56)	0.20 (0.03;0.64)	0.901
OD__OCTA_Macula_6.4×6.4__Perimeter	1.78 (0.73;2.92)	1.76 (0.65;3.13)	0.900
OS__OCTA_Macula_6.4×6.4__FAZ.Area	0.20 (0.03;0.53)	0.20 (0.02;0.57)	0.873
OS__OCTA_Macula_6.4×6.4__Perimeter	1.74 (0.71;2.83)	1.73 (0.61;2.95)	0.782
OU__OCTA_Macula_6.4×6.4__FAZ.Area	0.21 (0.04;0.55)	0.20 (0.04;0.59)	0.932
OU__OCTA_Macula_6.4×6.4__Perimeter	1.76 (0.72;2.88)	1.74 (0.79;2.95)	0.809
**OCT MACULA thickness (Thk) (microns, µm)**			
OD__OCT_Macula_Thk_ILM_BRM.um.__All.field.	298 (137;563)	299 (182;329)	0.669
OS__OCT_Macula_Thk_ILM_BRM.um.__All.field.	299 (178;330)	299 (222;464)	0.682
OU__OCT_Macula_Thk_ILM_BRM.um.__All.field.	299 (217;563)	299 (234;383)	0.639
**OCT MACULA volume (Vol, mm^3^)**			
OD__OCT_Macula_Vol_ILM_BRM.mm.3.__All.field.	31.3 (14.4;59.1)	31.4 (19.2;34.5)	0.609
OS__OCT_Macula_Vol_ILM_BRM.mm.3.__All.field.	31.5 (18.7;34.7)	31.4 (23.4;48.7)	0.645
OU__OCT_Macula_Vol_ILM_BRM.mm.3.__All.field.	31.5 (22.8;59.1)	31.4 (24.6;40.2)	0.629
**OCT DISC thickness (Thk) (microns, µm) measured within the slab of internal limiting membrane (ILM) to nerve fiber layer (NFL)**			
OD__OCT_Disc_Cube_6×6__Thk_ILM_NFL.all_sectors.um.	93.6 (0.00;142)	93.1 (0.00;128)	0.589
OS__OCT_Disc_Cube_6×6__Thk_ILM_NFL.all_sectors.um.	93.0 (32.5;764)	92.2 (31.5;205)	0.435
OU__OCT_Disc_Cube_6×6__Thk_ILM_NFL.all_sectors.um.	93.2 (42.2;427)	92.3 (15.8;153)	0.510
**OCT DISC volume (Vol, mm^3^)**			
OD__OCT_Disc_Cube_6×6__Rim.Volume.mm.3.	0.25 (0.00;1.03)	0.23 (0.00;1.27)	0.242
OS__OCT_Disc_Cube_6×6__Rim.Volume.mm.3.	0.24 (0.00;1.52)	0.24 (0.03;1.67)	0.092
OU__OCT_Disc_Cube_6×6__Rim.Volume.mm.3.	0.24 (0.02;1.03)	0.24 (0.06;1.39)	0.280

Legend: *p*, statistical *p*-value of Wilcoxon rank sum test, considered statistically significant when *p* < 0.05.

**Table 3:** Publication database review.

PubMed Search Term	No. Search Hits Total	No. Search Hits 2019	No. Search Hits 2020	No. Search Hits 2021	No. Search Hits 2022-10
COVID	304,677	50	91,258	136,733	107,167
COVID + eye	3686	0	1114	1710	1286
COVID + retina	527	0	135	246	208
COVID + retina + vessel	80	0	6	46	40

Legend: No., number of; PubMed, https://pubmed.ncbi.nlm.nih.gov, accessed on 2 November 2022.

**Table 4:** Overview of the literature.

OCTA Macula: Superficial Capillary Plexus (SRCP)		OCTA Macula: Deep Capillary Plexus (DRCP)		OCTA Disc: Radial Peripapillary Capillary (RPC), Layer: RNFL to ILM	
Sig. Parameter	Publication (COVID-19 Cohort Size)	Sig. Parameter	Publication (COVID-19 Cohort Size)	Sig. Parameter	Publication (COVID-19 Cohort Size)
Whole image (WI)	Ergul et al. 2022 (*n* = 32) (PMID: 35597442) [42] Cennamo et al. 2021 (*n* = 40) (PMID: 33781767) [18] Abrishami et al. 2021 (*n* = 31) (PMID: 33249111) [40]	Whole image (WI)	Abrishami et al. 2022 (*n* = 18) (PMID: 34636996) [43] Cennamo et al. 2021 (*n* = 40) (PMID: 33781767) [18] Abrishami et al. 2021 (*n* = 31) (PMID: 33249111) [40]	Whole image (WI)	Cennamo et al. 2021 (*n* = 40) (PMID: 33781767) [18]
WI hemifields	Aydemir et al. 2021 (*n* = 39) (PMID: 34587629) [44] Abrishami et al. 2021 (*n* = 31) (PMID: 33249111) [40]	WI hemifields	Abrishami et al. 2021 (*n* = 31) (PMID: 33249111) [40]	Small Vessels Inside Disc	Abrishami 2021 (*n* = 25) (PMID: 34840682) [45]
Fovea	Ergul et al. 2022 (*n* = 32) (PMID: 35597442) [42] Abrishami et al. 2021 (*n* = 31) (PMID: 3324911) [40]	Fovea	Ergul et al. 2022 (*n* = 32) (PMID: 35597442) [42] Cennamo et al. 2021 (*n* = 40) (PMID: 33781767) [18] Abrishami et al. 2021 (*n* = 31) (PMID: 33249111) [40]	Small Vessels Peripapillary (whole)	Abrishami 2021 (*n* = 25) (PMID: 34840682) [45]
Parafovea	Ergul et al. 2022 (*n* = 32) (PMID: 35597442) [42] Aydemir et al. 2021 (*n* = 39) (PMID: 34587629) [44] Abrishami et al. 2021 (*n* = 31) (PMID: 33249111) [40]	Parafovea	Abrishami et al. 2022 (*n* = 18) (PMID: 34636996) [43] Cennamo et al. 2021 (*n* = 40) (PMID: 33781767) [18] Abrishami et al. 2021 (*n* = 31) (PMID: 33249111) [40]	Small Vessels Peripapillary (sectoral)	Abrishami 2021 (*n* = 25) (PMID: 34840682) [45]
Perifovea	Ergul et al. 2022 (*n* = 32) (PMID: 35597442) [42] Abrishami et al. 2022 (*n* = 18) (PMID: 34636996) [43]	Perifovea	Abrishami et al. 2022 (*n* = 18) (PMID: 34636996) [43]		
		FAZ area/FAZ FD-300	Ergul et al. 2022 (*n* = 32) (PMID: 35597442) [42]		

Legend: sig., statistically significant with a significance level *p* < 0.05.

## Data Availability

Data not published in this manuscript are not publicly available due to privacy restrictions.
